# Increased hexosamine biosynthetic pathway flux alters cell–cell adhesion in INS-1E cells and murine islets

**DOI:** 10.1007/s12020-023-03412-9

**Published:** 2023-06-12

**Authors:** Dario Domenico Lofrumento, Alessandro Miraglia, Velia La Pesa, Antonella Sonia Treglia, Marcello Chieppa, Francesco De Nuccio, Giuseppe Nicolardi, Claudia Miele, Francesco Beguinot, Corrado Garbi, Bruno Di Jeso

**Affiliations:** 1 DiSTeBA, Centro Ecotekne, Strada Monteroni, University of Salento, 73100 Lecce, Italy; 2 Institute of Experimental Neurology and Division of Neuroscience, Neuropathology Unit, IRCCS San Raffaele Scientific Institute, 20132 Milan, Italy; 3 CNR, IEOS and DiSMeT, Via S. Pansini 5, University “Federico II”, Naples, Italy; 4 Dip. Medicina Molecolare e Biotecnologie Mediche, Via S. Pansini 5, University “Federico II”, Naples, Italy

**Keywords:** E-cadherin, Murine islets, Pancreatic beta-cell

## Abstract

**Purpose:**

In type 2 Diabetes, β-cell failure is caused by loss of cell mass, mostly by apoptosis, but also by simple dysfunction (dedifferentiation, decline of glucose-stimulated insulin secretion). Apoptosis and dysfunction are caused, at least in part, by glucotoxicity, in which increased flux of glucose in the hexosamine biosynthetic pathway plays a role. In this study, we sought to clarify whether increased hexosamine biosynthetic pathway flux affects another important aspect of β-cell physiology, that is β-cell–β-cell homotypic interactions.

**Methods:**

We used INS-1E cells and murine islets. The expression and cellular distribution of E-cadherin and β-catenin was evaluated by immunofluorescence, immunohistochemistry and western blot. Cell–cell adhesion was examined by the hanging-drop aggregation assay, islet architecture by isolation and microscopic observation.

**Results:**

E-cadherin expression was not changed by increased hexosamine biosynthetic pathway flux, however, there was a decrease of cell surface, and an increase in intracellular E-cadherin. Moreover, intracellular E-cadherin delocalized, at least in part, from the Golgi complex to the endoplasmic reticulum. Beta-catenin was found to parallel the E-cadherin redistribution, showing a dislocation from the plasmamembrane to the cytosol. These changes had as a phenotypic consequence a decreased ability of INS-1E to aggregate. Finally, in ex vivo experiments, glucosamine was able to alter islet structure and to decrease surface abundandance of E-cadherin and β-catenin.

**Conclusion:**

Increased hexosamine biosynthetic pathway flux alters E-cadherin cellular localization both in INS-1E cells and murine islets and affects cell–cell adhesion and islet morphology. These changes are likely caused by alterations of E-cadherin function, highlighting a new potential target to counteract the consequences of glucotoxicity on β-cells.

## Introduction

In type 2 Diabetes, β-cell failure is caused by loss of cell mass, mostly by apoptosis, but also by simple dysfunction (downregulation of specific gene expression, decline of glucose-stimulated insulin secretion) [1, 2]. Apoptosis and dysfunction are caused, at least in part, by glucotoxicity, in which increased flux of glucose in the hexosamine biosynthetic pathway (HBP) plays a role [3, 4]. Increased flux in the HBP is very efficiently exerted by a glucosamine (GlcN) treatment. In adipocytes. GlcN is 40 times more potent than glucose in mediating insulin resistance [5]. In addition, GlcN directly and selectively enters the HBP at the level of GlcN-6-Phosphate. As such, GlcN is a useful tool for studying the effects of increased HBP flux.

We have shown that increased HBP flux elicited a dedifferentiating response in β-cells, including pancreatic and duodenal homeobox 1 (PDX1), insulin1 and glut2 genes [6], and, interestingly, inhibits differentiation also in another insulin-sensitive cell, the adipocyte [7]. However, it is well known that β-cells-β-cells interactions and communications are crucial for normal β-cell function. A prominent role in β-cell–β-cell communications is exerted by connexins that cluster at gap junction domains of the β-cell membrane [8–12].

However, structural interactions are important as well [11]. E-cadherin plays an important role in β-cell–β-cell interactions both in pancreas development in vivo [13] and, in vitro, in cell adhesion in monolayer cultures [14, 15] and pseudoislets formation [8]. E-cadherin expression at the surface of islet rat β-cells is controlled by secretagogues including glucose and correlates with insulin secretion [16]. The MIN6 cell line sub-clone (C3) has been identified with reduced glucose stimulated insulin secretion (GSIS) and down-regulated E-cadherin expression [17]. Moreover, in rat pancreatic islets, increased expression of the adherens proteins α- and β-catenin is correlated with increased GSIS [18]. Analogously, homologous and heterologous intercellular contacts have a significant impact on insulin secretion in human β-cells [19, 20]. Moreover, E-cadherin affects gap junction communication, suggesting a cross-talk between the two type of junction [21, 22].

In this study we sought to clarify whether increased HBP flux affects β-cell–β-cell adhesion and if this may participate in β-cell dysfunction and altered islet physiology.

## Materials and methods

### Materials

Media, sera and antibiotics were purchased from Invitrogen (Paisley, UK). Chemicals were from Sigma- Aldrich (St Louis, MO, USA). Glucosamine was from Santa Cruz Biotechnology (Santa Cruz, CA, USA). Antibodies were anti-E-cadherin and anti-β-catenin (Cell Signaling, Danvers, MA, USA), anti-calnexin (Invitrogen, Waltham, USA), anti-β-actin (monoclonal; Sigma), anti-GS28 protein [23] (a gift of Dr. S. Bonatti). Collagenase P was from Roche Applied Science (Penzberg, Germany).

### Cell culture

The clonal beta cell line INS-1E was used between passages 54 and 95. INS-1E cells were cultured in a humidified atmosphere containing 5% (vol./vol.) CO_2_ in complete medium composed of RPMI 1640 supplemented with 5% (vol./vol.) heat-inactivated FCS, 1 mmol/l sodium pyruvate, 50 μmol/l 2-mercaptoethanol, 2 mmol/l glutamine, 10 mmol/l HEPES, 100 U/ml penicillin and 100 μg/ml streptomycin. The maintenance culture was split once a week and cells were seeded at 3 × 10^6^ cells/ 75 cm^2^ in Falcon bottles (BD Biosciences Labware, Franklin Lakes, NJ, USA). The potential presence of mycoplasma was regularly checked using a photometric enzyme immunoassay (Roche, Penzberg, Germany). For most experiments, INS-1E were seeded at 2 × 10^5^ cells/ml in Falcon 24 well plates and used 4 to 5 days thereafter, with one medium change on day 3 or 4.

### Immunofluorescence

1.5 × 10^5^ cells were plated on 12 mm diameter glass coverslips. 48 h later, cells were vehicle-treated or treated with 7.5 mM GlcN for 24 h. Cells were fixed for 20 min with 3% paraformaldehyde (Sigma) in PBS containing 0.9 mM CaCl_2_ and 0.5 mM MgCl_2_ (PBS-CM) at room temperature, washed twice in 50 mM NH_4_Cl in PBS-CM and twice in PBS-CM. Cells were permeabilized for 5 min in 0.5% Triton-X100 (Bio-Rad) in PBS-CM and incubated for 30 min in 0.5% gelatin (Sigma) in PBS-CM. Cells were then incubated for 1 h with the primary antibodies (anti-E-cadherin 1:100, anti- β-catenin 1:100) diluted in 0.5% BSA (Sigma) in PBS. After three washes with 0.2% gelatin in PBS-CM cells were incubated with the second primary antibodies (anti-calnexin 1:100, anti-GS28 1:50) for 1 h. After three washes with 0.2% gelatin in PBS-CM cells were incubated for 20 min with the appropriate rhodamine- or fluorescein-tagged goat anti-mouse or anti-rabbit antibody (Jackson ImmunoResearch, West Grove, PA), diluted 1:50 in 0.5% BSA in PBS. After final washes with PBS, the coverslips were mounted on a microscope slide and examined with a Zeiss 510 confocal laser scanning microscope. Samples were observed by three investigators, without knowledge of the experimental conditions.

### Immunohistochemistry

After being isolated, the islets, sedimented in an eppendorf tube, were fixed in 4% paraformaldehyde for 15 min. Subsequently, three washes in PBS and inclusion in 1% agarose in H_2_O were performed. The polymerized agarose was finally embedded in paraffin. Immunohistochemistry was performed following a standard avidin–biotin complex procedure. Briefly, serial sections were cleared of paraffin in xylene overnight, incubated in 0.3% hydrogen peroxide diluted in methanol for 1 h to inactivate endogenous peroxidases, rehydrated through graded alcohol baths and blocked in 5% normal goat serum, diluted in TBS, for 1 h. Subsequently, sections were incubated overnight with anti-E-cadherin at ratio of 1:100, or anti- β-catenin at a ratio of 1:100, both in 1% BSA diluited in TBS, at 4 °C. Finally, the slides were incubated with biotinylated secondary antibody at a 1:1000 diluition in 1% BSA in TBS, for 1 h at room temperature. After three 10-minute washes in TBS, to visualize the formation of the antigen-antibody complex, sections were incubated for 1 h with extravidin peroxidase diluted 1:1500 in 1% BSA in TBS, and color development was obtained with 3,3′-diaminobenzidine in presence of 2 mmol/L hydrogen peroxide. As a negative control, tissue slides were incubated with non-immune serum.

### Western blots analysis

Western blots were carried out as previously reported [24]. Briefly, cells were plated in 60 mm diameter plates to about 50% confluence 48 h before harvesting. After evaluation of protein content, 30 μg of cell extract was analyzed by SDS-PAGE and electrotransferred to polyvinylidene difluoride membrane. Blocking was for 15 h at 4 °C with Tris-buffered saline-Tween 20 (TBST) buffer (10 mM Tris [pH 8.0], 150 mM NaCl, 0.1% Tween 20) containing 10% nonfat dry milk, followed by incubation in TBST buffer for 2 h at room temperature with a 1:1000 dilution of anti-E-cadherin and anti-β-catenin, 1:5000 anti-β-actin. After being washed with TBST, the blot was incubated for 1 h at room temperature with antirabbit horseradish peroxidase-conjugated antibodies diluted 1:3000 in TBST. Band detection was by enhanced chemiluminescence. The molecular mass markers were from Sigma.

### Cell–cell adhesion assays

Cell–cell adhesion was examined by using the hanging-drop aggregation assay. Sub-confluent INS-1 cells were subjected to 5 mM and 7.5 mM GlcN treatments for 24 h. Cells were detached with 0.5% tripsin-EDTA and washed with PBS twice, then rendered into single-cell suspension by three gentle passes through a 27-gauge needle. The single-cell suspension of 1 × 10^4^ cells in 30 ml of solution was suspended as a hanging drop from the lid of a 24-well culture dish and allowed to aggregate overnight at 37 °C in 5% CO_2_ with humidity, either in presence or absence of GlcN. Samples were observed by three investigators, without knowledge of the experimental conditions.

### Islet isolation

Islets were isolated from 6-month-old C57Bl/6J mice. Animals were killed by cervical dislocation, the fur was soaked with ethanol and the abdomen was opened. The pancreas was inflated by KRBH injection and excised. The excised pancreas was washed twice with KRBH and digested with collagenase P in a water bath (37 °C), shaken by hand for 5–8 min. The digested pancreas was treated with Dnase I. The islets were handpicked under a stereo-microscope and cultured for 24 h in complete RPMI 1640 medium. They were treated with 5 mM GlcN beginning 24 h after isolation. Islets were observed by three investigators, without knowledge of the experimental conditions.

### Cell viability assay

The conversion of MTT (3-(4,5-dimethylthiazol-2-yl)- 2,5-diphenol tetrazolium bromide) by murine islet was used as an indicator of cell viability. Murine islets were isolated and treated with 5 mM GlcN for 72 h, as described above. After gentle centrifugation (100 × *g*, 5 min.) islets were incubated in 1 ml of culture medium plus MTT to a final concentration of 0.5 mg/ml. After a 3-h incubation, medium was removed and islets were lysed in acidified isopropanol/HCl 0.04 N. The lysates were subsequently read on a spectrophotometer at 550 nm (Bio-rad, Richmond, CA, USA). The results were expressed as percent viability compared to control.

## Results

### Following GlcN treatment total E-cadherin expression did not change but there was an increase of E-cadherin intracellular pool

**Fig. 1:**
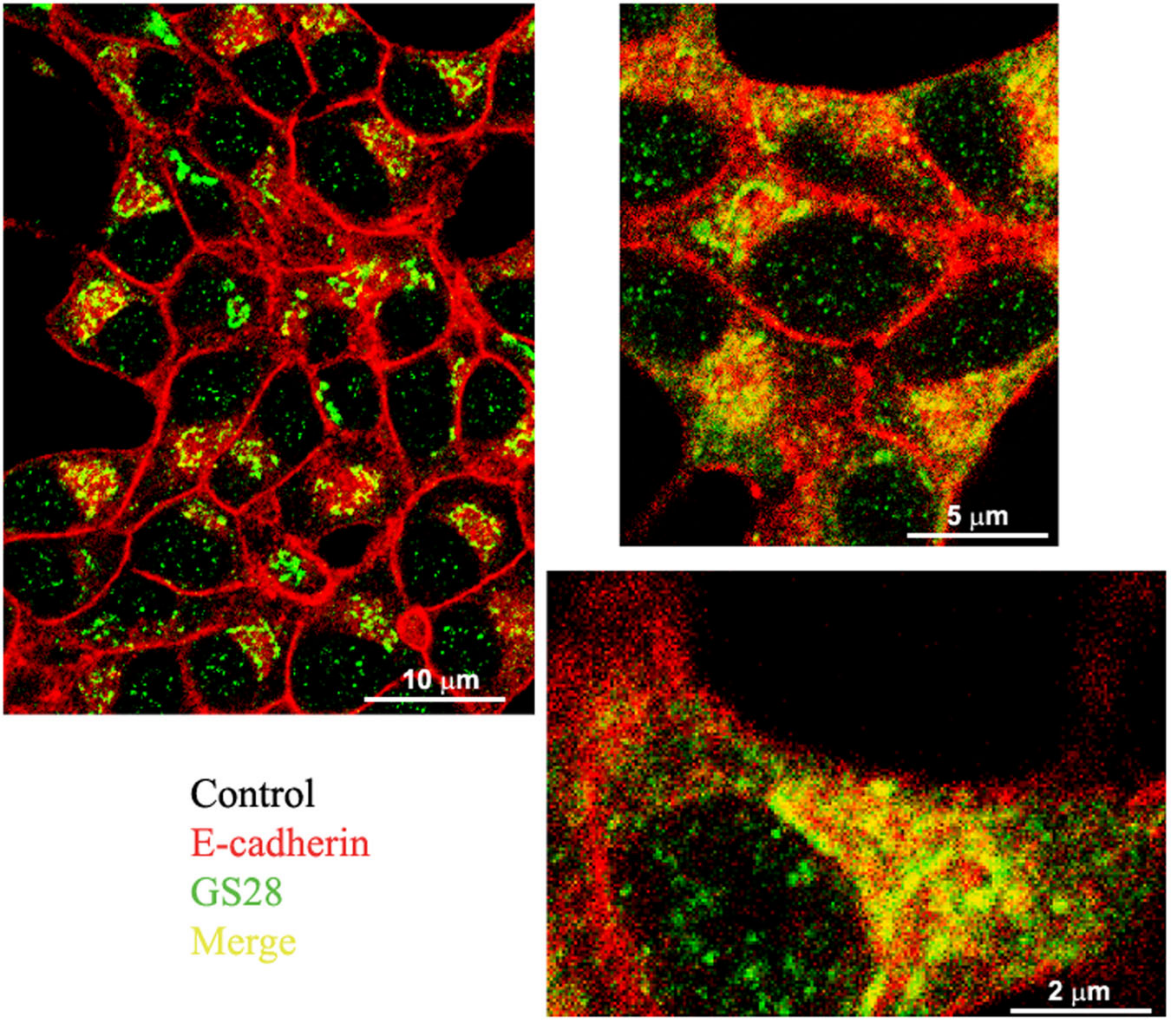
In normal conditions E-cadherin is localized mostly on plasmamembrane with a minor intracellular pool localizing in the Golgi. INS-1E cells were grown on glass coverslips for 48 h, then were vehicle-treated for 24 h. Cells were double-stained with anti-E-cadherin and anti-GS28 (a Golgi marker) antibodies. *n* = 3

**Fig. 2:**
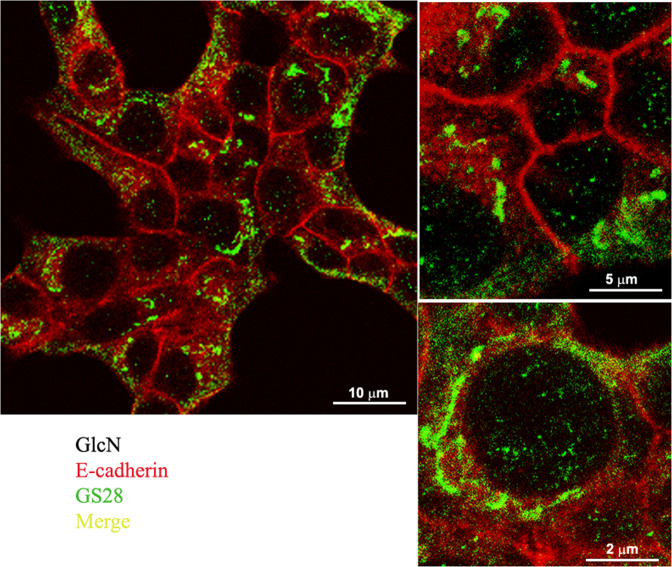
Following GlcN treatment there was a decrease of the plasmamembrane localization, a gain of a diffuse intracellular and a loss of the Golgi localization. INS-1E cells were grown on glass coverslips for 48 h, then were treated with 7.5 mM GlcN for 24 h. Cells were double-stained with anti-E-cadherin and anti-GS28 (a Golgi marker) antibodies. Following GlcN treatment there was a decrease of the plasmamembrane localization, a gain of a diffuse intracellular and a loss of the Golgi localization. *n* = 3

GlcN elicits a dedifferentiating response in INS-1E cells (decreased expression of *Ins1*, *Glut2*, and *Pdx1* genes), profoundly inhibits GSIS, while does not affect β-cell viability [6]. In this study we sought to clarify whether GlcN affects β-cell–β-cell adhesion. We first checked if GlcN decreased the expression of E-cadherin. Contrary to our expectations, GlcN, at various concentration, including that able to inhibit differentiation and GSIS in INS-1E cells (7.5 mM) [6], did not change E-cadherin expression (Online Resources 1). Next, we evaluated the hypothesis that GlcN changed E-cadherin cellular distribution. We studied the cellular distribution of E-cadherin by immunofluorescence. In normal conditions E-cadherin is localized mostly on plasmamembrane with a minor intracellular pool localizing, in part, in the Golgi, as evidenced by some overlaps between E-cadherin and GS28 (a Golgi marker) signals (Fig. 1). Following a GlcN treatment there was a loss of the plasmamembrane E-cadherin staining and a gain of a diffuse intracellular staining. As a result, there was a loss of overlap between E-cadherin and GS28 (Fig. 2, and Online Resources 2, 3, 4 vs 5, 6, and 7). Next, we sought to clarify, at least partially, the intracellular site of localization of E-cadherin following GlcN treatment. Thus, we doubly stained cells with E-cadherin and calnexin, a marker of the ER. As shown in Online Resources 8, 9, 10 vs 11, 12, 13, and 14, while in control condition there was no overlap between E-cadherin and calnexin, following GlcN treatment such an overlap appeared, although it was partial.

These data indicate that following a GlcN treatment E-cadherin decreased at the plasmamembrane and the intracellular E-cadherin pool shifted from the Golgi to, in part, the ER.

### Following GlcN treatment the total amount of β-catenin decreased and the cytosolic β-catenin increased

**Fig. 3:**
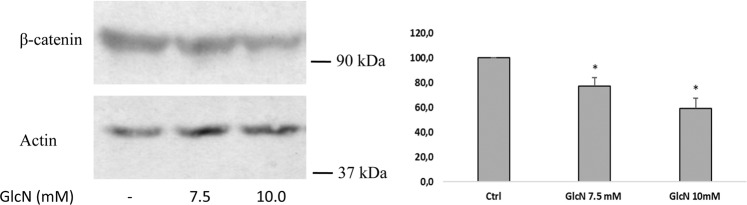
Following GlcN treatment the total amount of β-catenin decreased. Sub-confluent INS-1E cells were mock treated or treated with 7.5 mM GlcN for 48 h. Cell extracts were evaluated for protein content and resolved by SDS-PAGE, blotted, and probed for β-catenin and actin. Following GlcN treatment total β-catenin decreased. The graph represents the media ± SD of three independent experiments. The difference between treated and control cells was evaluated using Student’s *t* test. *p* < 0.05 was considered significant. *p < 0.05. *n* = 3

**Fig. 4:**
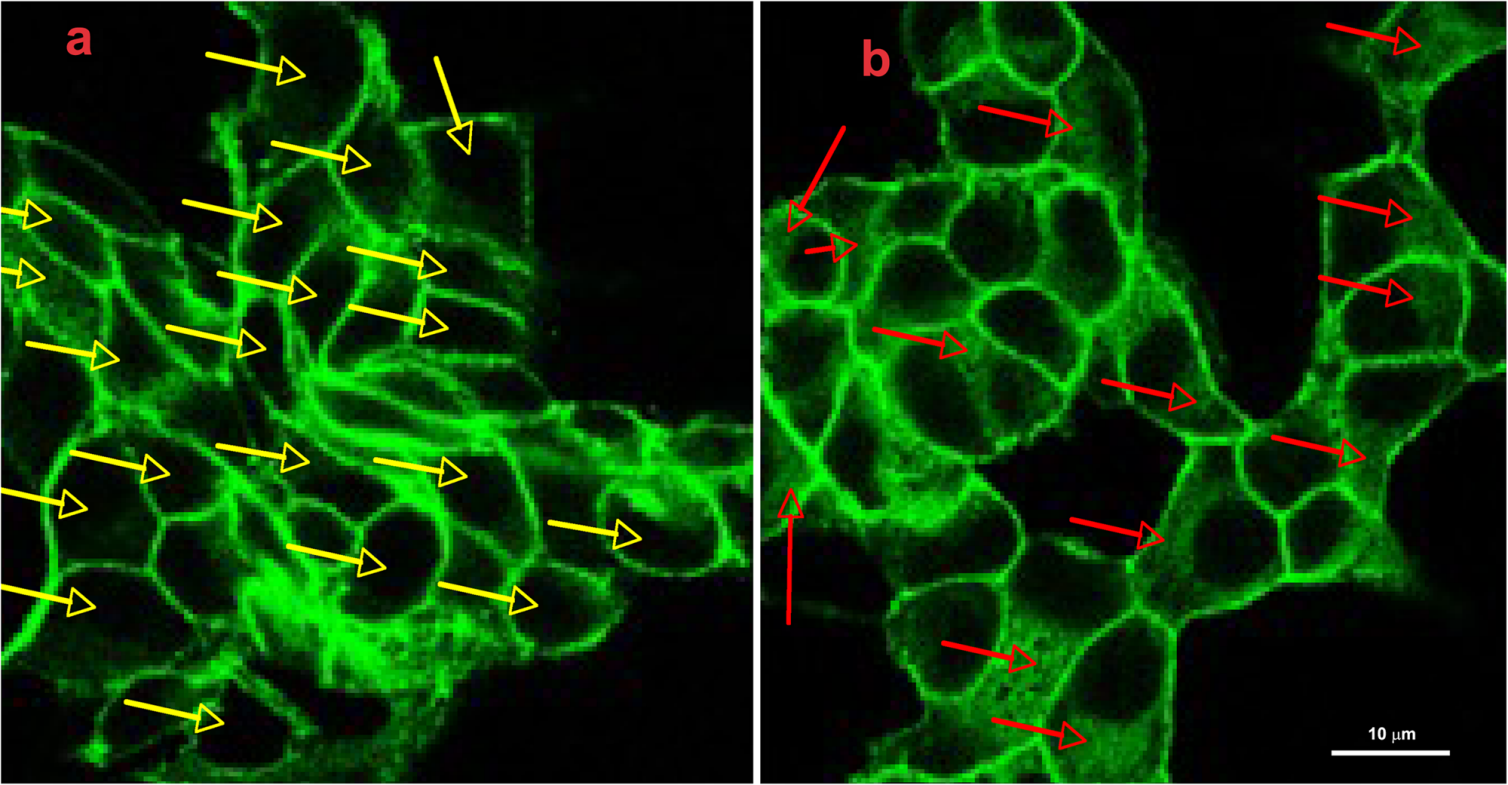
Following GlcN treatment cytosolic β-catenin increased. INS-1E cells were grown on glass coverslips for 48 h, then were vehicle-treated (**a**) or treated with 7.5 mM GlcN for 24 h (**b**). Cells were stained with anti-β-catenin antibodies. Yellow arrows in the control cells indicate cells without intracellular β-catenin, red arrows in treated cells indicate cells with intracellular β-catenin. Following GlcN treatment there was the appearance of intracellular β-catenin. *n* = 2

Since E-cadherin interacts through a core region of 30 amino acids within its cytoplasmic C-terminus domain with β-catenin [25], we analyzed the total amount of β-catenin after GlcN treatment. β-catenin slightly decreased following a GlcN treatment (Fig. 3). Next, we sought to determine if GlcN also changed the cellular distribution on β-catenin. Thus, we performed immunofluorescence studies. By immunofluorescence, following a GlcN treatment there was the appearance of β-catenin in the cytosol, with a high percentage of cells becoming positive for cytosolic β-catenin (Fig. 4a, b, arrows).

### GlcN decreased cell–cell adhesion

**Fig. 5:**
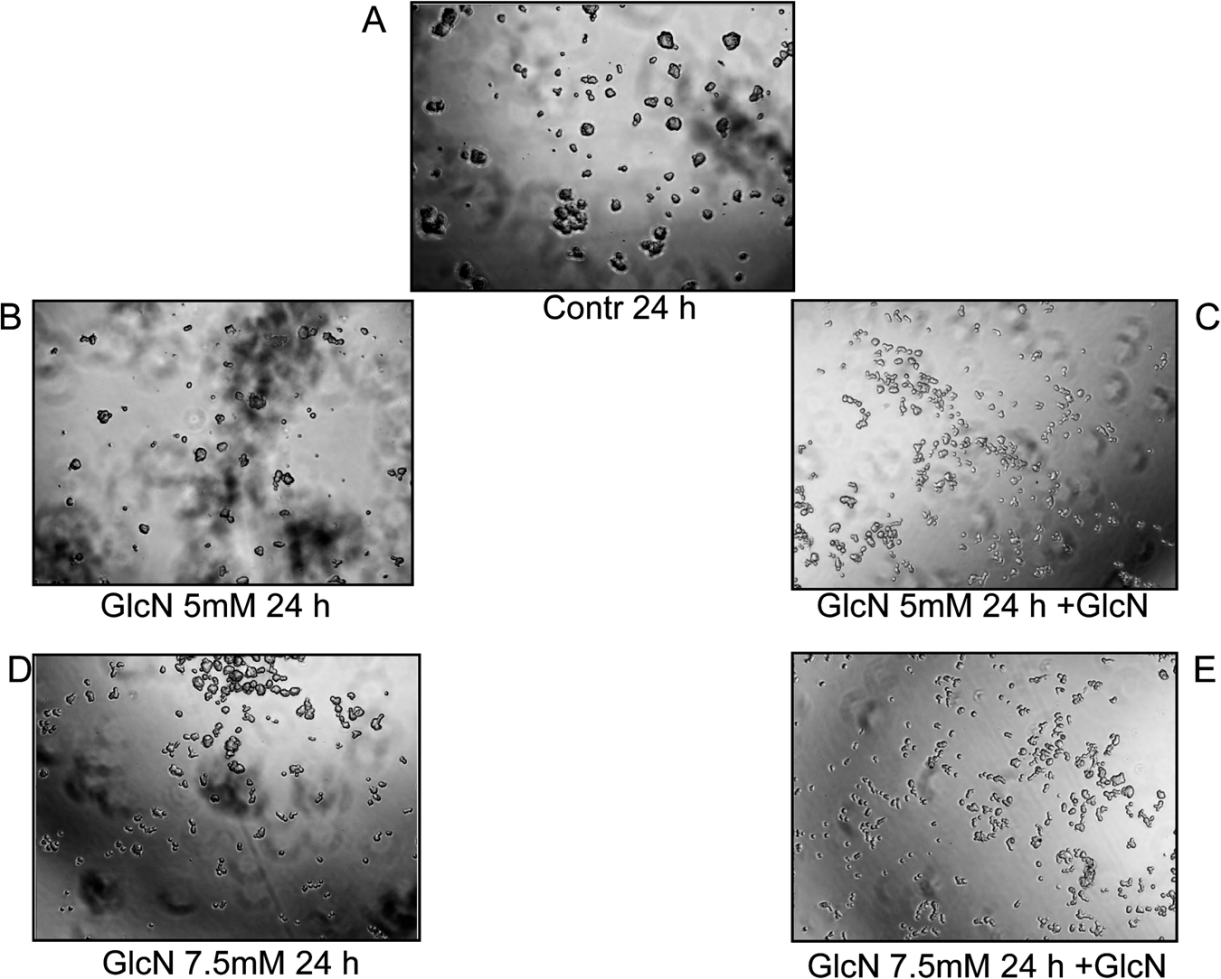
Following GlcN treatment there was a decrease of cell-cell adhesion. Sub-confluent INS-1E cells were either mock treated (control, **A**) or subjected to 5 mM (**B**, **C**) and 7.5 mM GlcN treatments for 24 h (**D**, **E**). Then were subjected to the hanging-drop aggregation assay as outlined in Materials and Methods, either in presence (+GlcN, **C**, **E**) or absence of GlcN (**B**, **D**). GlcN caused a dramatic decrease of cell-cell adhesion. *n* = 3

We reasoned that the above-described changes in E-cadherin and β-catenin cellular distribution induced by GlcN could have a phenotypic impact, specifically on the strength of cell–cell interaction. To verify this hypothesis, INS-1E cells treated or not treated with GlcN were subjected to the hanging drop assay in the presence of Ca^+2^ to evaluate cadherin-dependent cell–cell adhesion. As shown in Fig. 5, 5 and 7.5 mM GlcN decreased cadherin-dependent cell-cell adhesion, suggesting that the above-described changes produced a functional defect of E-cadherin.

Taken togheter, these data suggest alterations in the E-cadherin/β-catenin complexes that affect cell-cell adhesions. Interestingly, at the same concentrations and times used here, GlcN inhibits GSIS, without affecting cell viability [6].

### GlcN altered islet structure

**Fig. 6:**
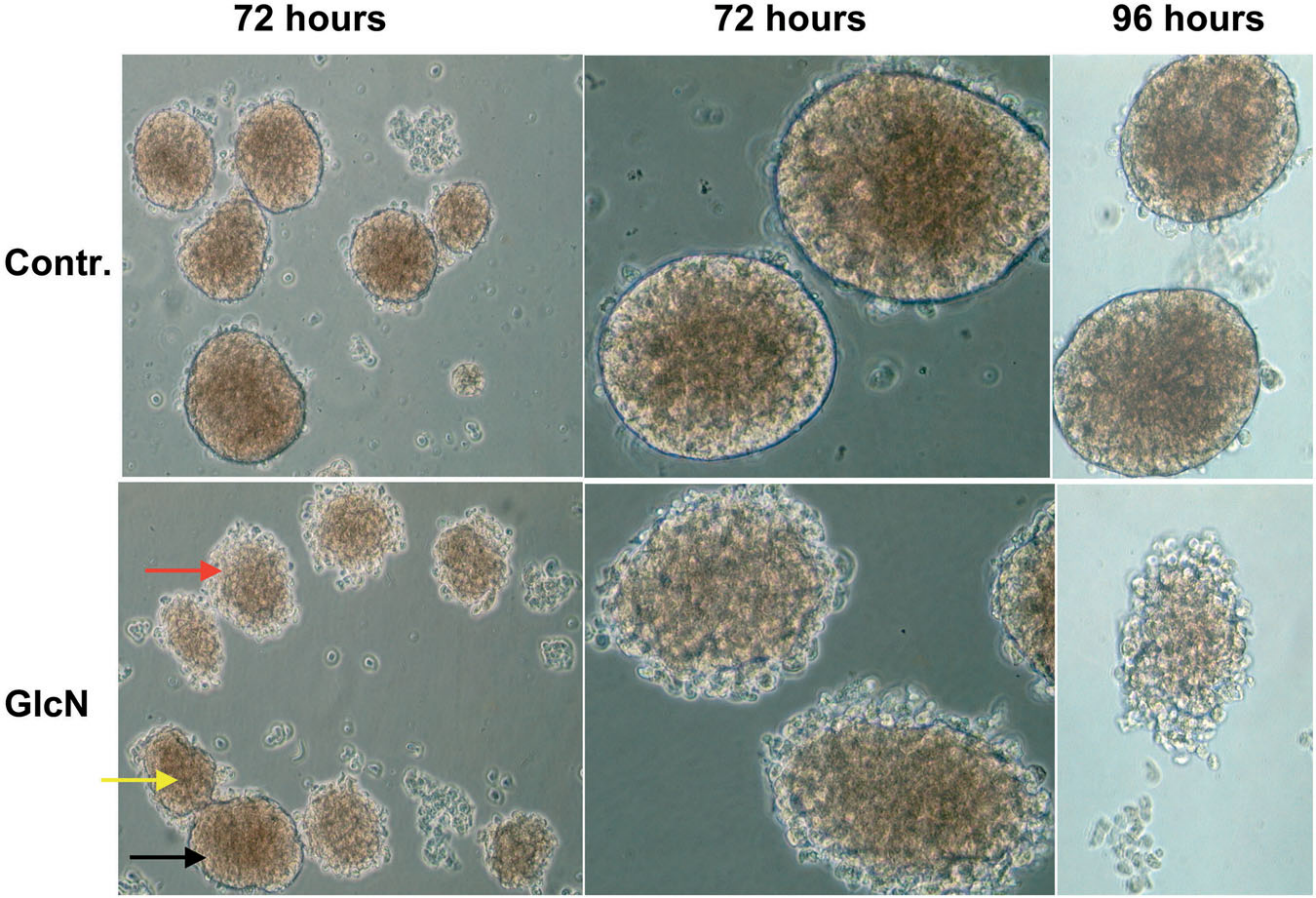
Following GlcN treatments there was an alteration of islet structure. Murine islets were isolated as outlined in Materials and methods. They were treated with 5 mM GlcN beginning 24 h after isolation, for 72 and 96 h. Islets were observed and photographed by three investigators, without knowledge of the experimental conditions. Black arrow indicates an insula preserving a normal morphology after GlcN treatment, yellow arrow indicates an insula with initial alterations of morphology, red arrow indicates an insula with an higher degree of alteration of morphology. *n* = 4

**Fig. 7:**
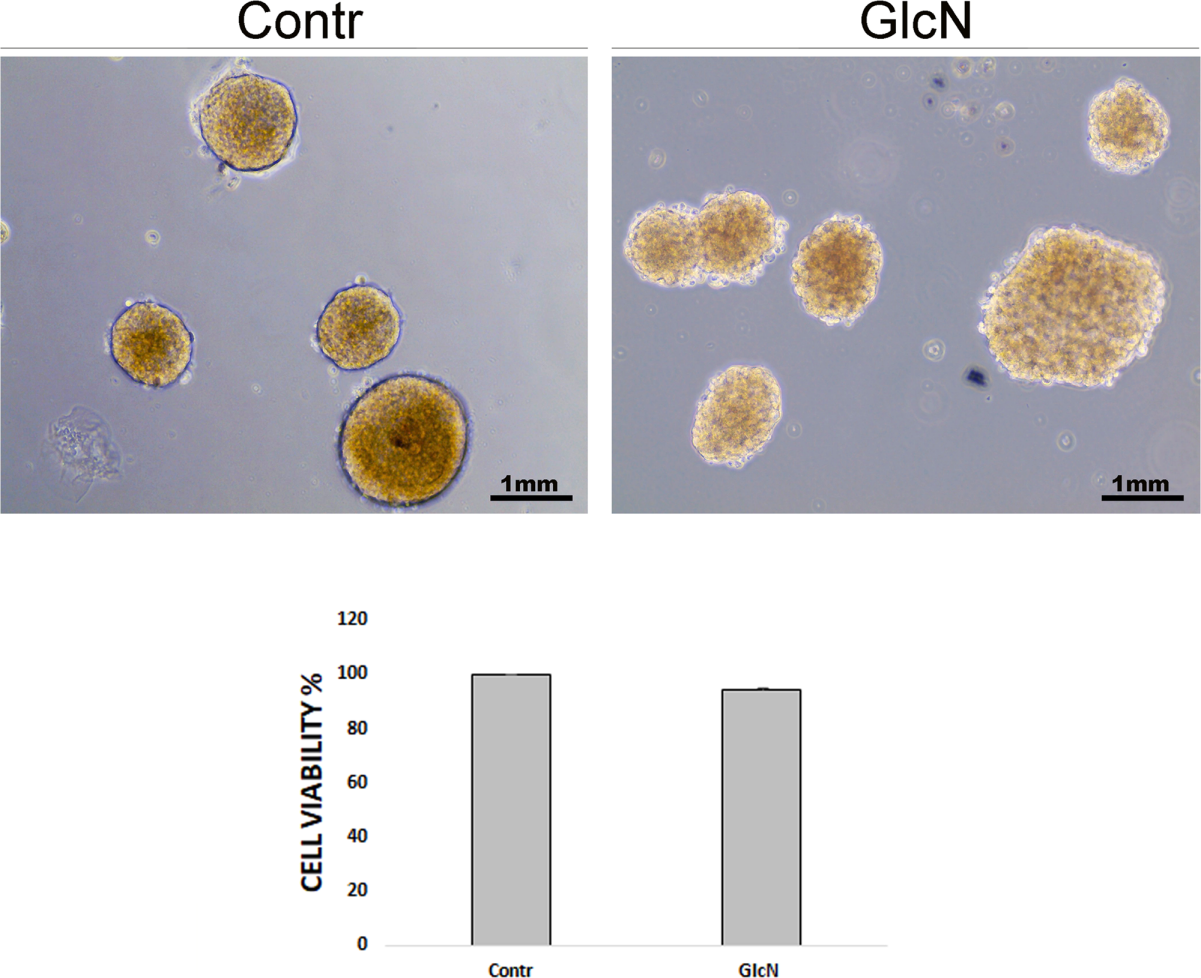
Following GlcN treatment viability of islet cells was not altered. Murine islets were isolated as outlined in Materials and methods. They were treated with 5 mM GlcN beginning 24 h after isolation, for 72 h. Islets were observed and photographed (upper panels) by three investigators, without knowledge of the experimental conditions. After a gentle centrifugation were subjected to MTT assay. The treatment did not decrease cell viability (lower panel) (while decreased islet aggregation, upper panels). The graph represents the media ± SD of three independent experiments. The difference between treated and control cells was evaluated using Student’s *t* test. *p* < 0.05 was considered significant. *n* = 3. There was a very slight difference between control and treated islets. *p* = 0.13

**Fig. 8:**
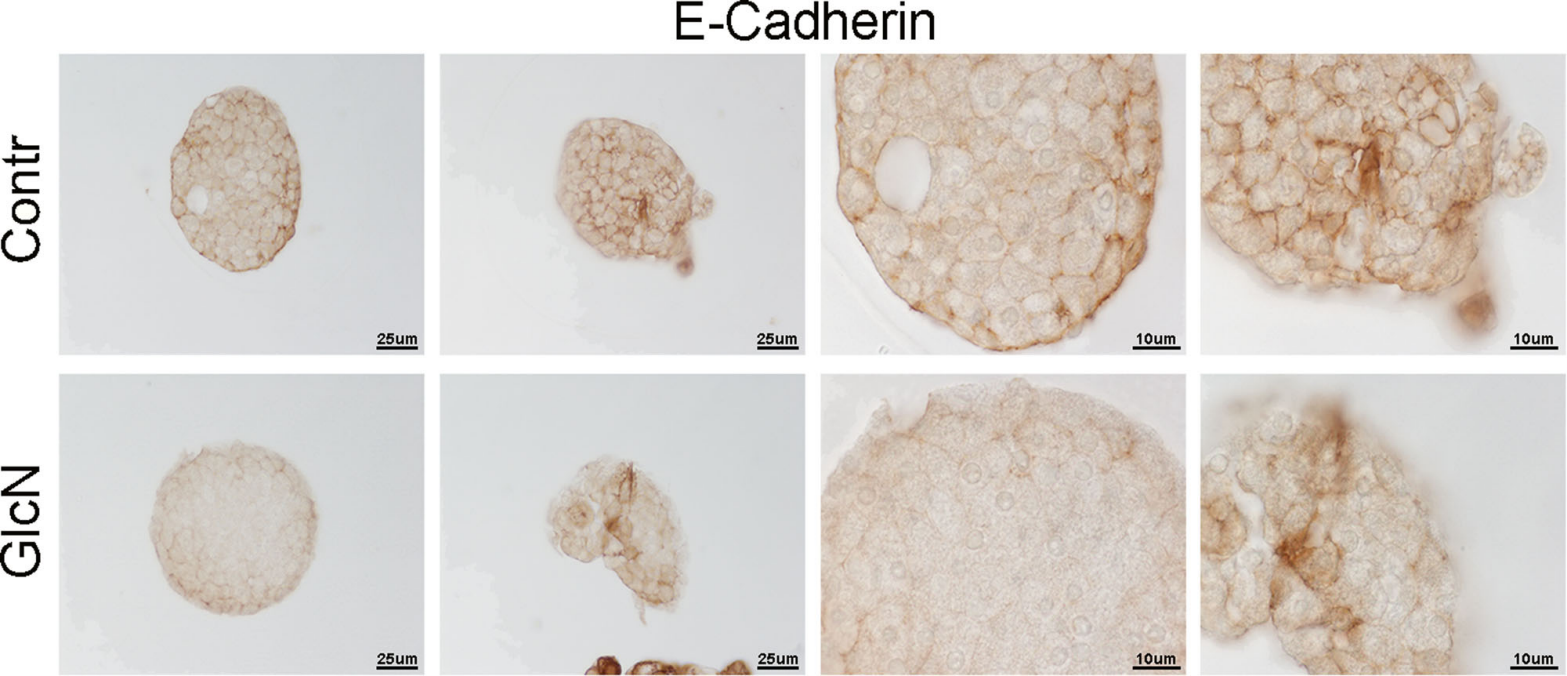
Following GlcN treatment of islet the abundance of plasmamembrane E-cadherin decreased. Murine islets were isolated as outlined in Materials and methods. They were treated with 5 mM GlcN beginning 24 h after isolation, for 48 h (the experimental condition associated to islet ER stress, dedifferentiation, and beta cell functional impairment [6]). Islets were embedded in paraffin and subjected to immunohistochemistry as outlined in “Materials and methods”. *n* = 3

**Fig. 9:**
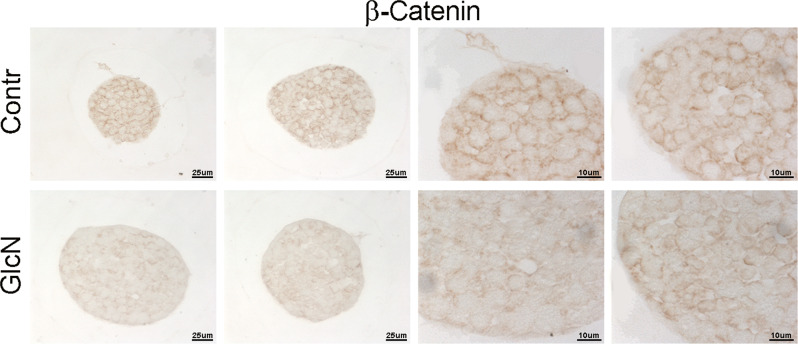
Following GlcN treatment of islet the abundance of plasmamembrane β-catenin decreased. Murine islets were isolated as outlined in Materials and methods. They were treated with 5 mM GlcN beginning 24 h after isolation, for 48 h h (the experimental condition associated to islet ER stress, dedifferentiation, and beta cell functional impairment [6]). Islets were embedded in paraffin and subjected to immunohistochemistry as outlined in “Materials and methods”. *n* = 3

Next, we sought to verify if the GlcN effects on INS-1E cells could be present in ex vivo experiments. Thus, murine islets were isolated and treated with GlcN as outlined in “Materials and methods”. As shown in Fig. 6, GlcN treatment caused an alteration of islet structure, most evident at islet periphery, where it assumed the features of structure disaggregation. In Fig. 6 is present the sequence of morphologic stages leading to disruption of islet architecture, probably reflecting different sensitivity of different islet to the GlcN effect. Thus, the black arrow indicates an insula preserving a normal morphology after GlcN treatment, the yellow arrow indicates an insula with initial alteration of morphology, and the red arrow indicates an insula with an higher degree of alteration of morphology. Next, we sought to exclude that the observed islet cell disaggregation after 72 h exposure to GlcN might be due to cell death, by performing viability assays with MTT. Thus, islets were isolated, treated with GlcN for 72 h, photographed (Fig. 7, upper panels), gently sedimented and subjected to MTT assay. As shown in Fig. 7, lower panel, treatment with GlcN for 72 h, did not decrease cell viability (while affected islet aggregation, upper panel). Finally, we sought to investigate E-cadherin and β-catenin abundance at the plasmamembrane also in islet cells. To do this, we adopted, a GlcN treatment of 48 h (the experimental condition associated to islet ER stress, dedifferentiation, and beta cell functional impairment [6]), and an immunohstochemistry approach, given the difficulty/inability of islets to attach to glass coverslips. As shown in Figs. 8 and 9, GlcN caused a dramatic decrease of E-cadherin (Fig. 8) and β-catenin (Fig. 9) abundance at the plasmamembrane of islet cells.

## Discussion

In this study we have examined the effects of increased HBP flux by GlcN on the cellular distribution and function of E-cadherin in INS-1E cells. We show that GlcN alters the intracellular distribution of E-cadherin and β-catenin. Moreover, the cadherin-dependent cell-cell adhesion is dramatically decreased. Finally, the structure of murine islets is altered, the plasmamembrane abundance of E-cadherin and β-catenin is decreased in islet cells, but cell viability is preserved.

Islets of Langerhans play a crucial role in regulating glucose metabolism and their function is crucially dependent on the islet cell-islet cell contacts. Dispersed cells poorly respond to glucose for insulin secretion but their responsiveness can be restored upon reaggregation of the islet cells [26, 27]. This integrated response effect has been ascribed to the function of two family of proteins, cadherins and connexins. Intra-islet cell communication is crucial in coordinating appropriate release of insulin and is largely achieved through connexins (gap-junction) mediated communication [8–12].

However, also adherens junctions-mediated contacts play a role. E-cadherin is the principal adhesion molecule found in the insulin-secreting islet β-cells and plays a pivotal role in both acquisition and maintenance of cell-cell contacts and islet architecture. Thus, E-cadherin is crucial both in pancreas development in vivo [13] and, in vitro, in cell adhesion in monolayer cultures [14, 15] and pseudoislets formation [8]. In addition, a close relationship is present between E-cadherin expression and glucose-stimulated insulin secretion. E-cadherin expression at the surface of islet rat β-cells correlates with insulin secretion [16]. The MIN6 cell line sub-clone (C3) has been identified with reduced glucose evoked insulin secretion and down-regulated E-cadherin expression [17]. The decline of glucose-induced insulin secretion that follows islet dispersal is coupled with decreased E-cadherin expression [28]. While it is not clear if the contribution of E-cadherin to insulin secretion is a direct effect on the secretory process or is secondary to its effect on islet architecture, nevertheless, alterations in E-cadherin expression and/or function impact on β-cell integrated response for insulin secretion.

Hyperglycemia has several negative consequences on β-cell function. Hyperglycemia and increased HBP flux by GlcN have been reported to play a role in beta-cell dysfunction through multiple mechanisms. Thus, glucotoxic β-cells show decreased expression of several β-cell specific genes such as insulin, GLUT2, glucokinase [6, 29–33]. In addition, the glucotoxic β-cells show impaired secretory vesicle fusion [34]. Finally, since secreted insulin has a positive effect on insulin transcription [35, 36], the glucotoxic effects may interrupt this positive feedback mechanism. In this study we verified the hypothesis that increased HBP flux may negatively impact on β-cell function through an effect on E-cadherin function. We show that a treatment of INS-1E cells with GlcN change the intracellular distribution of E-cadherin, increasing the intracellular pool. Moreover, the intracellular pool also changed its organelle localization, from a Golgi localization to, at least partial, ER localization. This may be related to decreased ER to Golgi transport secondary to ER stress [37, 38], and, indeed, we have previously shown that GlcN at the same concentrations used here is able to cause ER stress in INS-1E cells [6]. This is not surprising as β-cells are highly susceptible to ER stress since, like thyroid cells [38, 39] (another endocrine cell), have a high protein load, synthesizing large quantities of (a single) protein (insulin and thyroglobulin [40–44], respectively). Proinsulin represents up to 20% of the total mRNA and 30–50% of the total protein synthesis of the β-cell [45]. We have also found a redistribution of β-catenin from the plasmamembrane to the cytosol paralleling the E-cadherin redistribution, indicating that E-cadherin and β-catenin redistribute together from plasmamembrane to intracellular pools. This suggests that GlcN caused a loss of β-catenin-mediated E-cadherin connection to the actin cytoskeleton.

In line with these considerations, the above-described alterations of E-cadherin and β-catenin intracellular distribution have a negative effect on β-cell–β-cell adhesion. Thus, cell–cell adhesion is dramatically decreased. This defect well correlates with the already reported negative effect of GlcN (at the same concentrations and timing) on glucose-regulated insulin secretion [6]. GlcN treatment caused an alteration of islet structure, most evident at islet periphery, where assumed the features of structure disaggregation. By comparing islet structure at 72 and 96 h treatment (Fig. 6), we suggest that islet architecture alteration progress with a periphery-center dynamic, in line with a progressive diffusion of GlcN towards the islet center. However, under the same conditions, GlcN did not decrease islet cell viability (Fig. 7). Finally, GlcN, under the experimental conditions associated to islet ER stress, dedifferentiation, and beta cell functional impairment [6]), caused a dramatic decrease of E-cadherin (Fig. 8) and β-catenin (Fig. 9) abundance at the plasmamembrane of islet cells.

In conclusion, in this study we have described a new mechanism by which glucotoxicity may affect insulin secretion, by affecting E-cadherin function, highlighting a new potential target to counteract the consequences of glucotoxicity on β-cells.

## Supplementary information

Online Resource Legends

Online Resource 1

Online Resource 2

Online Resource 3

Online Resource 4

Online Resource 5

Online Resource 6

Online Resource 7

Online Resource 8

Online Resource 9

Online Resource 10

Online Resource 11

Online Resource 12

Online Resource 13

Online Resource 14
