# Supplementary material for: Increased hexosamine biosynthetic pathway flux alters cell–cell adhesion in INS-1E cells and murine islets
Source: Endocrine. 2023 Jun 12;81(3):492–502. doi: 10.1007/s12020-023-03412-9 (PMC10403402; doi:10.1007/s12020-023-03412-9)
Supplement: Supplementary file 1 — Online Resource Legends [file 12020_2023_3412_MOESM1_ESM.doc]

**Online Resource Legends.**

**Online Resources 1** Following GlcN treatment total E-cadherin expression did not change

Sub-confluent INS-1E cells were mock treated or treated with increasing concentrations of GlcN for 48 hours. Cell extracts were evaluated for protein content, resolved by SDS-PAGE, blotted, and probed for E-cadherin and actin.

**Online Resources 2, 3, 4, 5, 6, 7** **Following GlcN treatment there was a decrease of the plasmamembrane localization, a gain of a diffuse intracellular and a loss of the Golgi localization of E-cadherin**

INS-1E cells were grown on glass coverslips for 48 hours, then were vehicle-treated (2-4) or treated with 7.5 mM GlcN (5-7) for 24 hours. Cells were double-stained with anti-E-cadherin and anti-GS28 (a Golgi marker) antibodies.

**Online Resources 8, 9, 10, 11, 12, 13, 14 Following GlcN treatment there was a gain of ER localization of E-cadherin** INS-1E cells were grown on glass coverslips for 48 hours, then were vehicle-treated (8-10) or treated with 7.5 mM GlcN (12-14) for 24 hours. Cells were double-stained with anti-E-cadherin and anti-calnexin (an ER marker) antibodies.
