# Supplementary figures and images for: Increased hexosamine biosynthetic pathway flux alters cell–cell adhesion in INS-1E cells and murine islets
Source: Endocrine. 2023 Jun 12;81(3):492–502. doi: 10.1007/s12020-023-03412-9 (PMC10403402; doi:10.1007/s12020-023-03412-9)

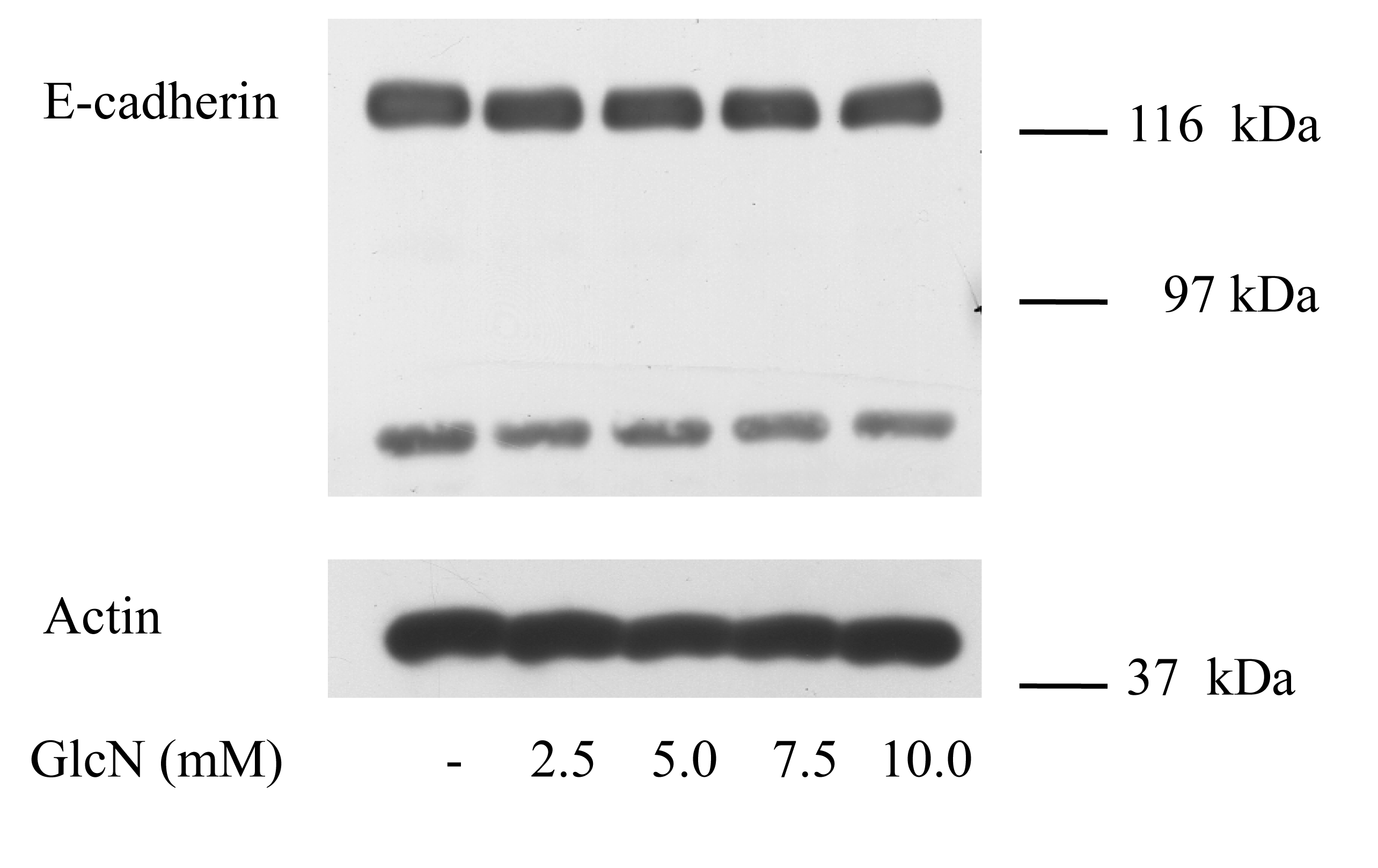

Supplement: Supplementary file 2 — Online Resource 1 [file 12020_2023_3412_MOESM2_ESM.tif]

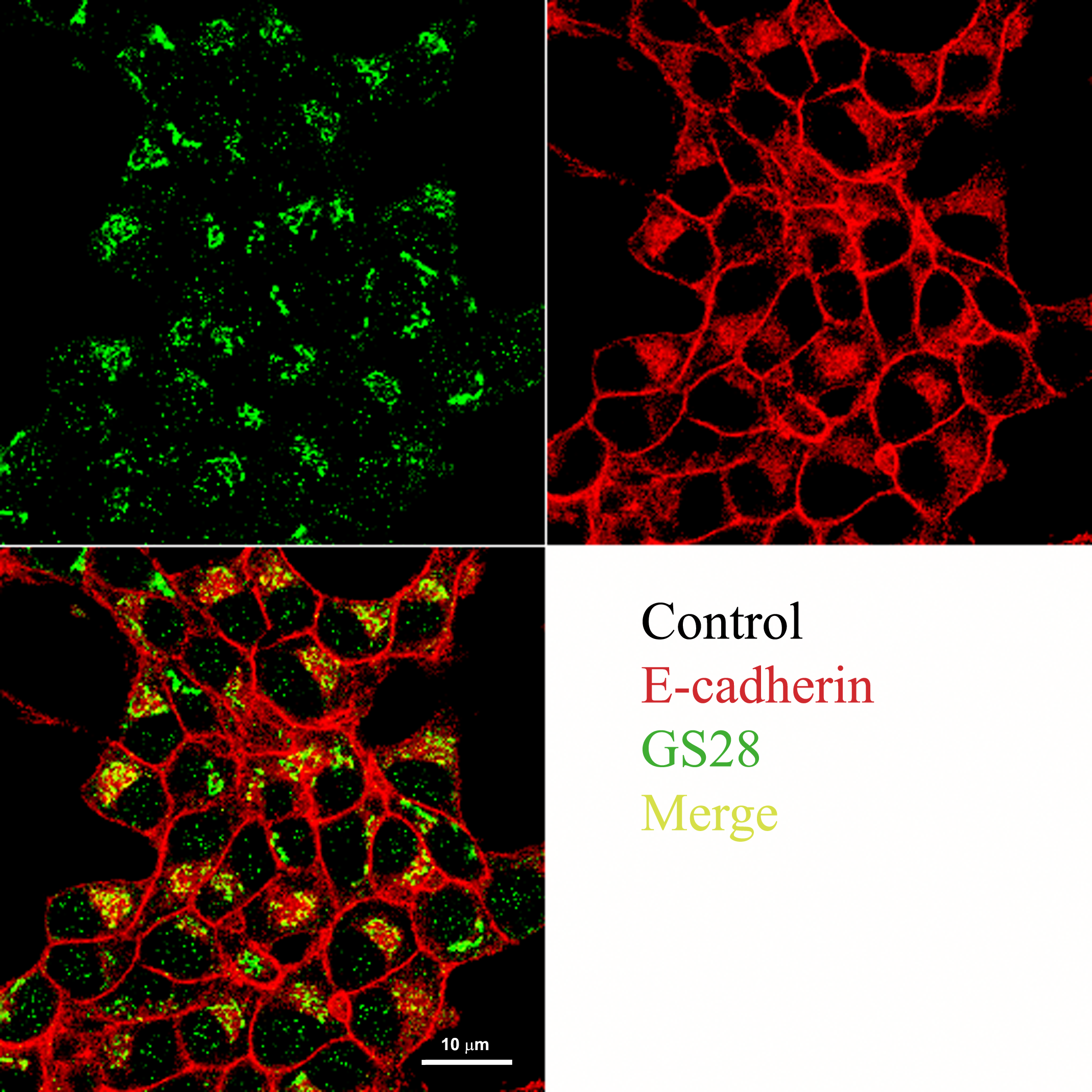

Supplement: Supplementary file 3 — Online Resource 2 [file 12020_2023_3412_MOESM3_ESM.tif]

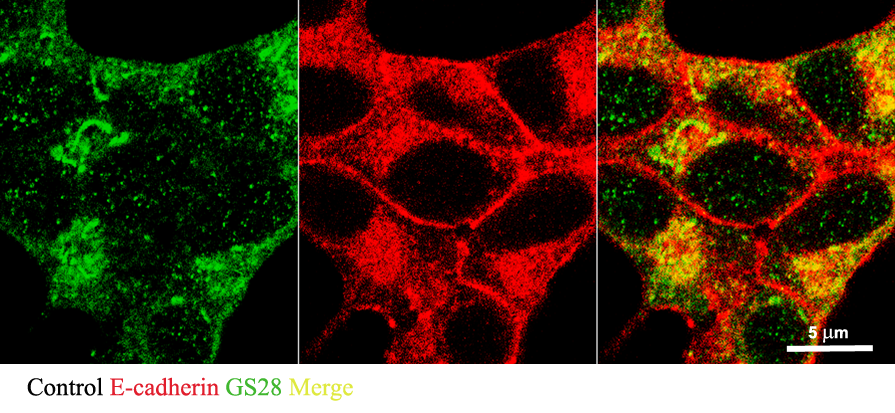

Supplement: Supplementary file 4 — Online Resource 3 [file 12020_2023_3412_MOESM4_ESM.tif]

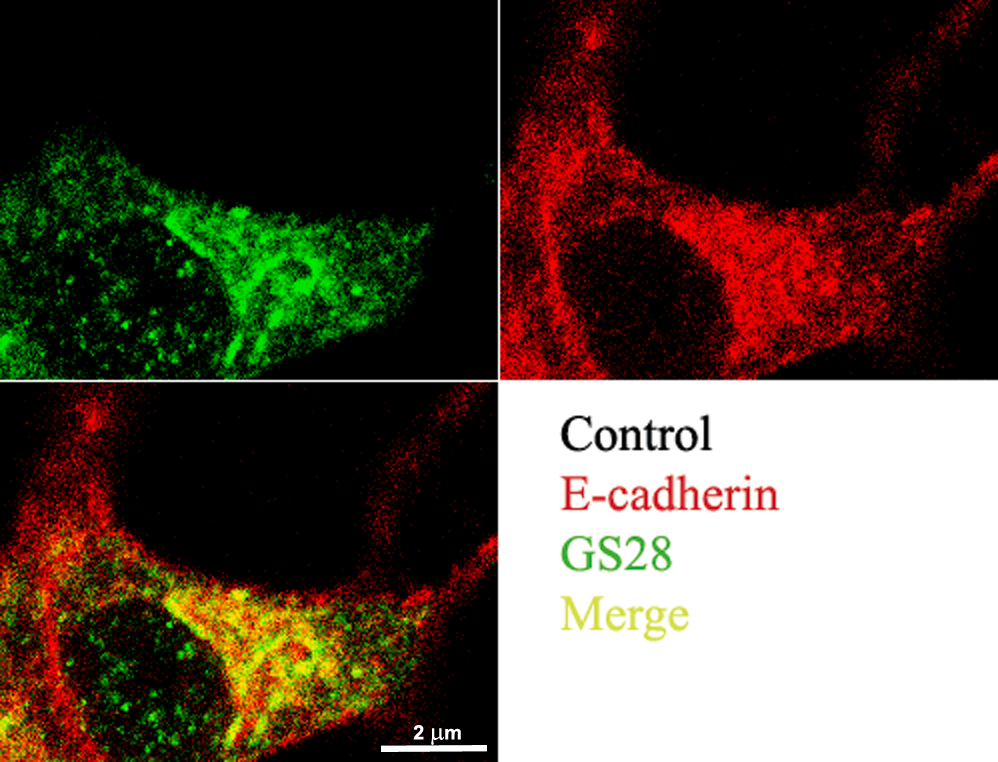

Supplement: Supplementary file 5 — Online Resource 4 [file 12020_2023_3412_MOESM5_ESM.tif]

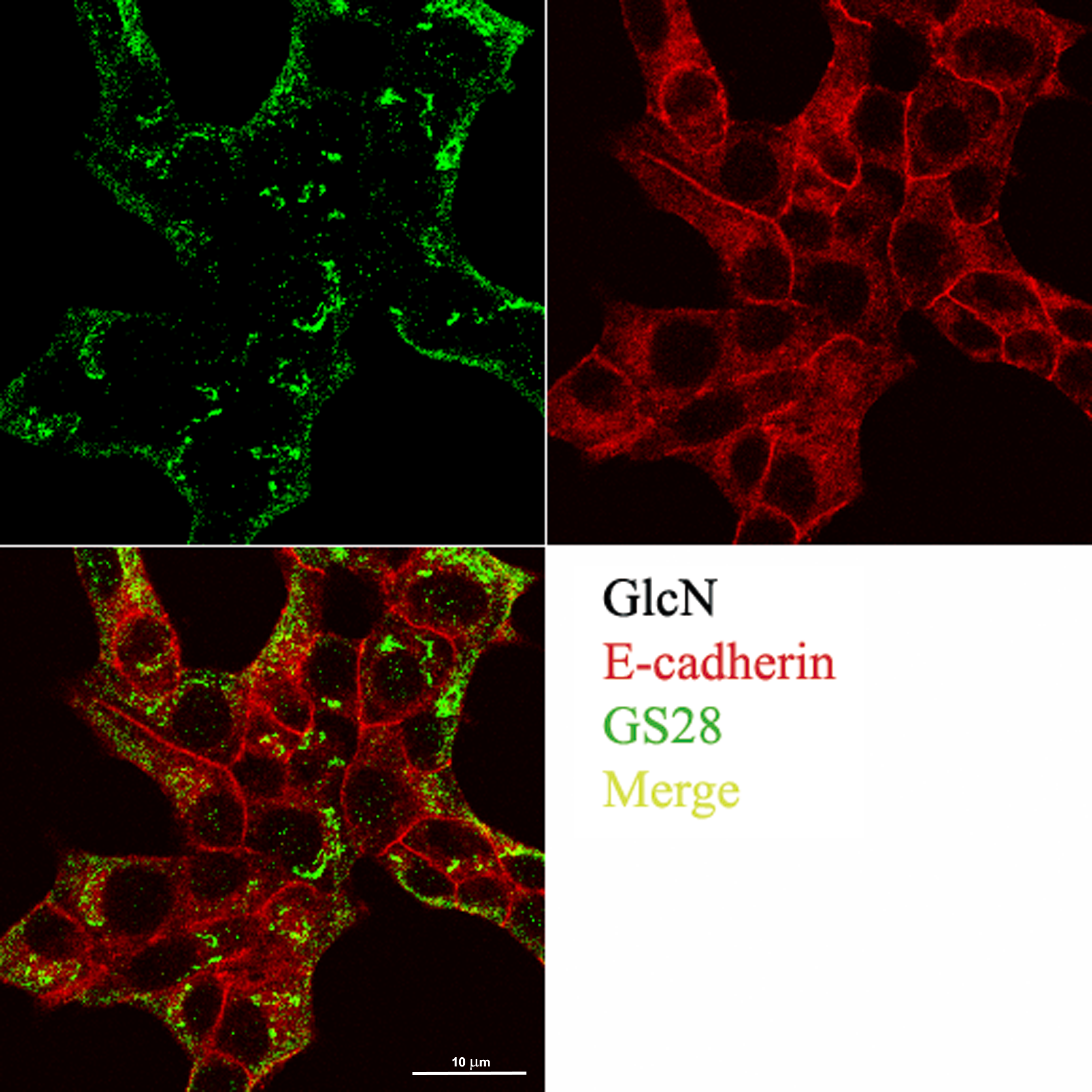

Supplement: Supplementary file 6 — Online Resource 5 [file 12020_2023_3412_MOESM6_ESM.tif]

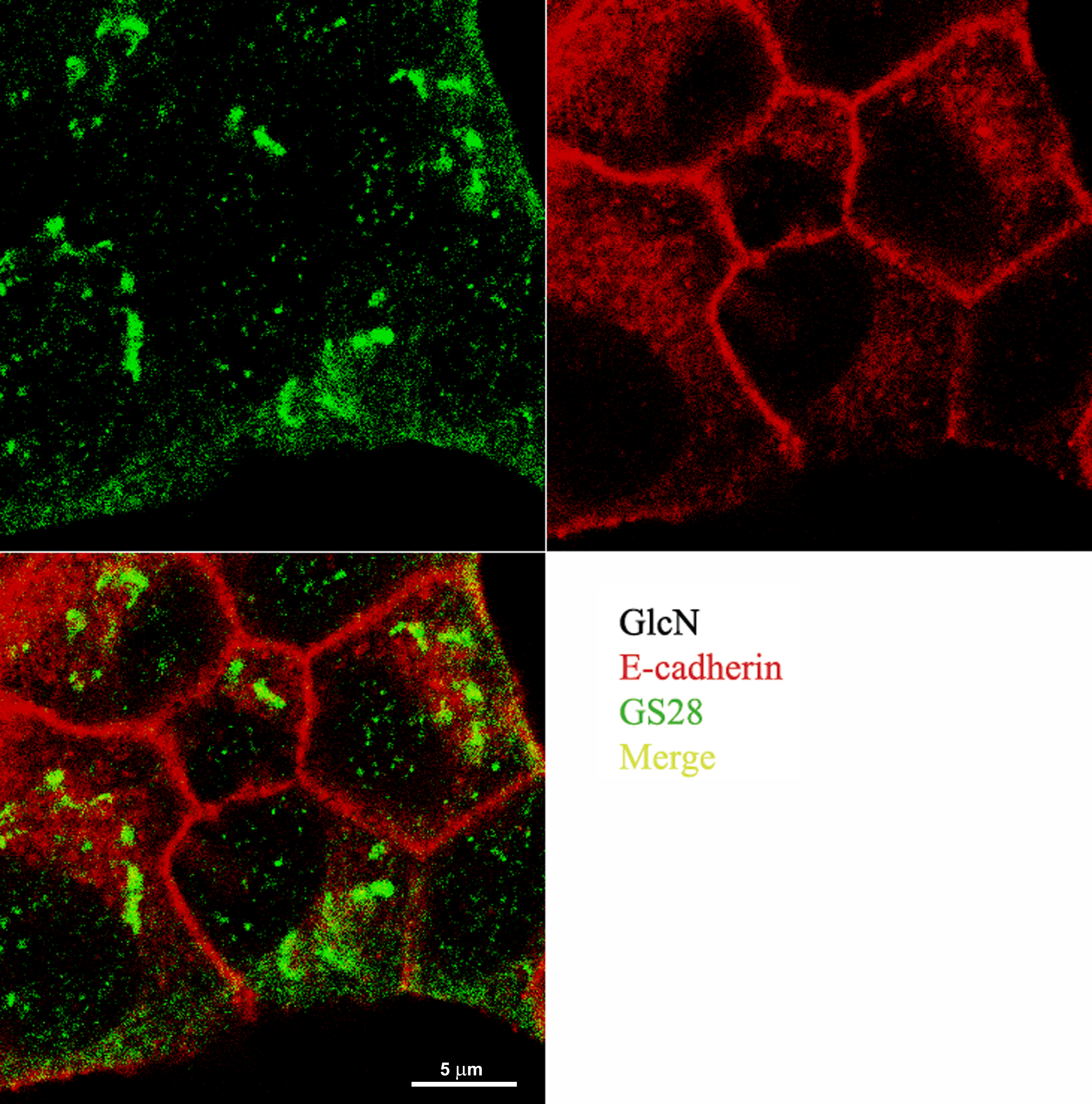

Supplement: Supplementary file 7 — Online Resource 6 [file 12020_2023_3412_MOESM7_ESM.tif]

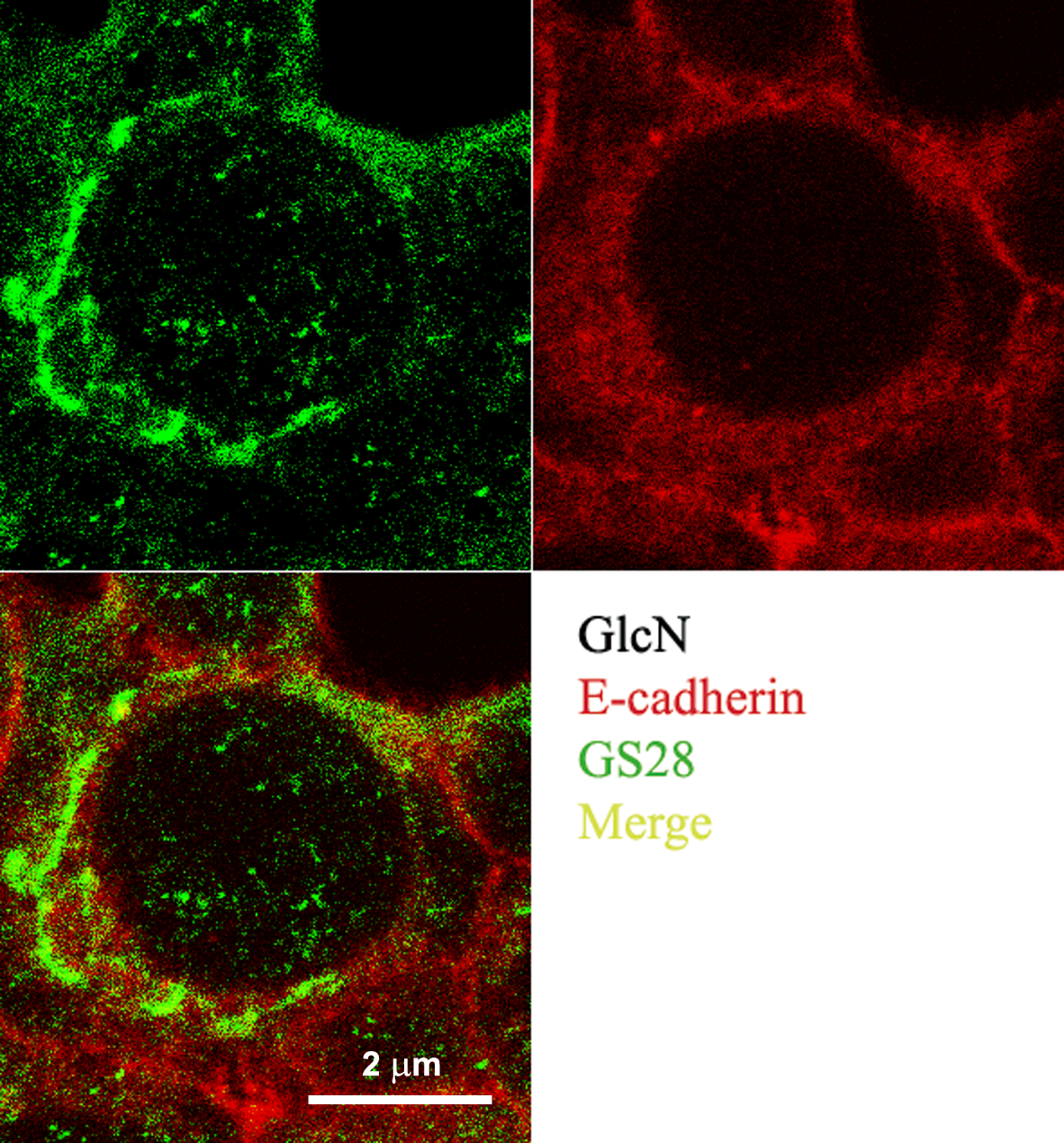

Supplement: Supplementary file 8 — Online Resource 7 [file 12020_2023_3412_MOESM8_ESM.tif]

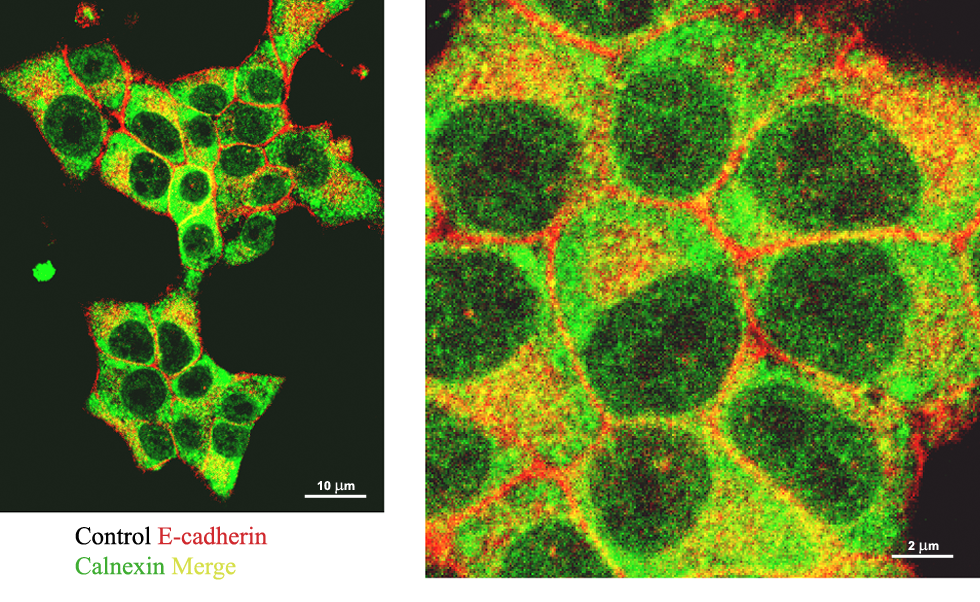

Supplement: Supplementary file 9 — Online Resource 8 [file 12020_2023_3412_MOESM9_ESM.tif]

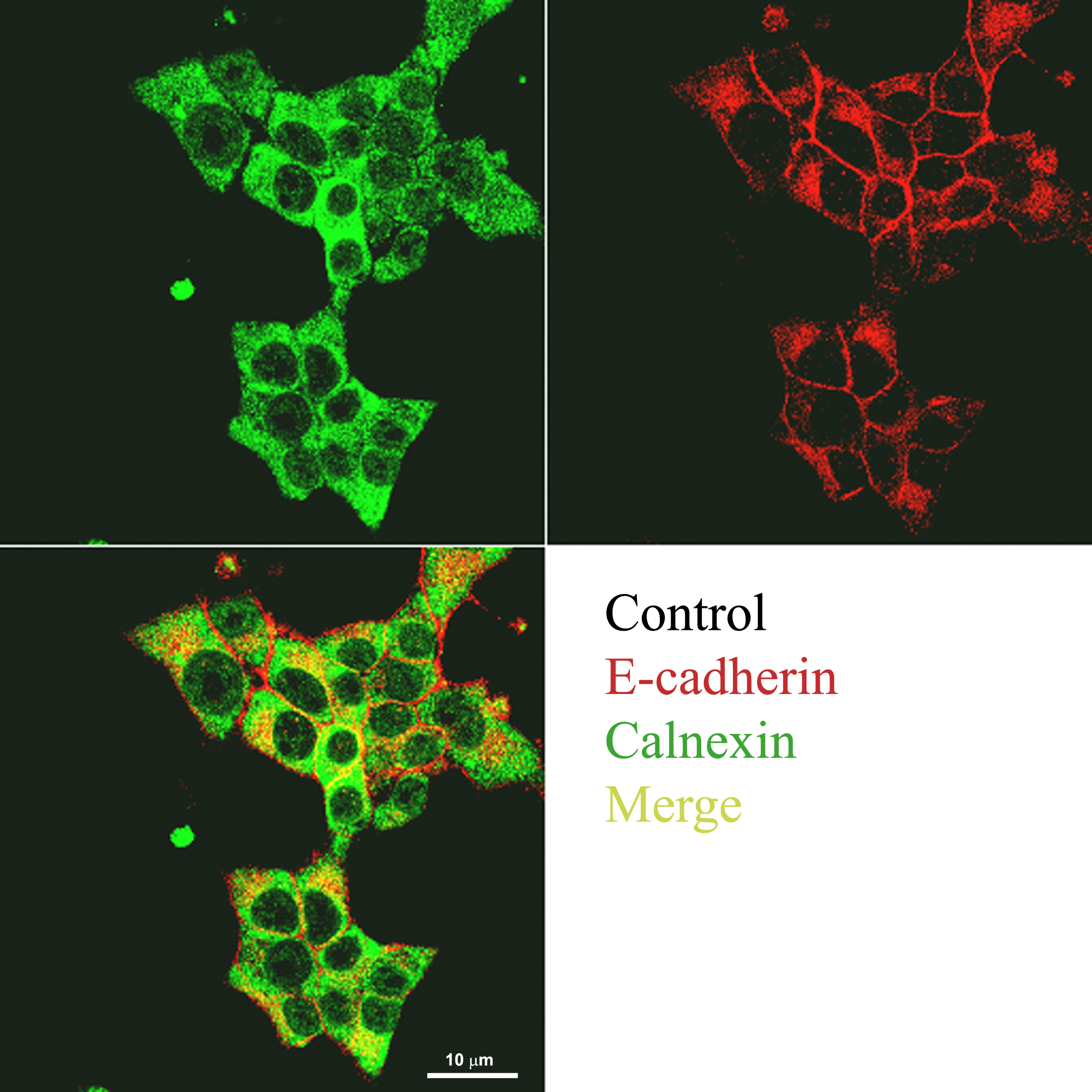

Supplement: Supplementary file 10 — Online Resource 9 [file 12020_2023_3412_MOESM10_ESM.tif]

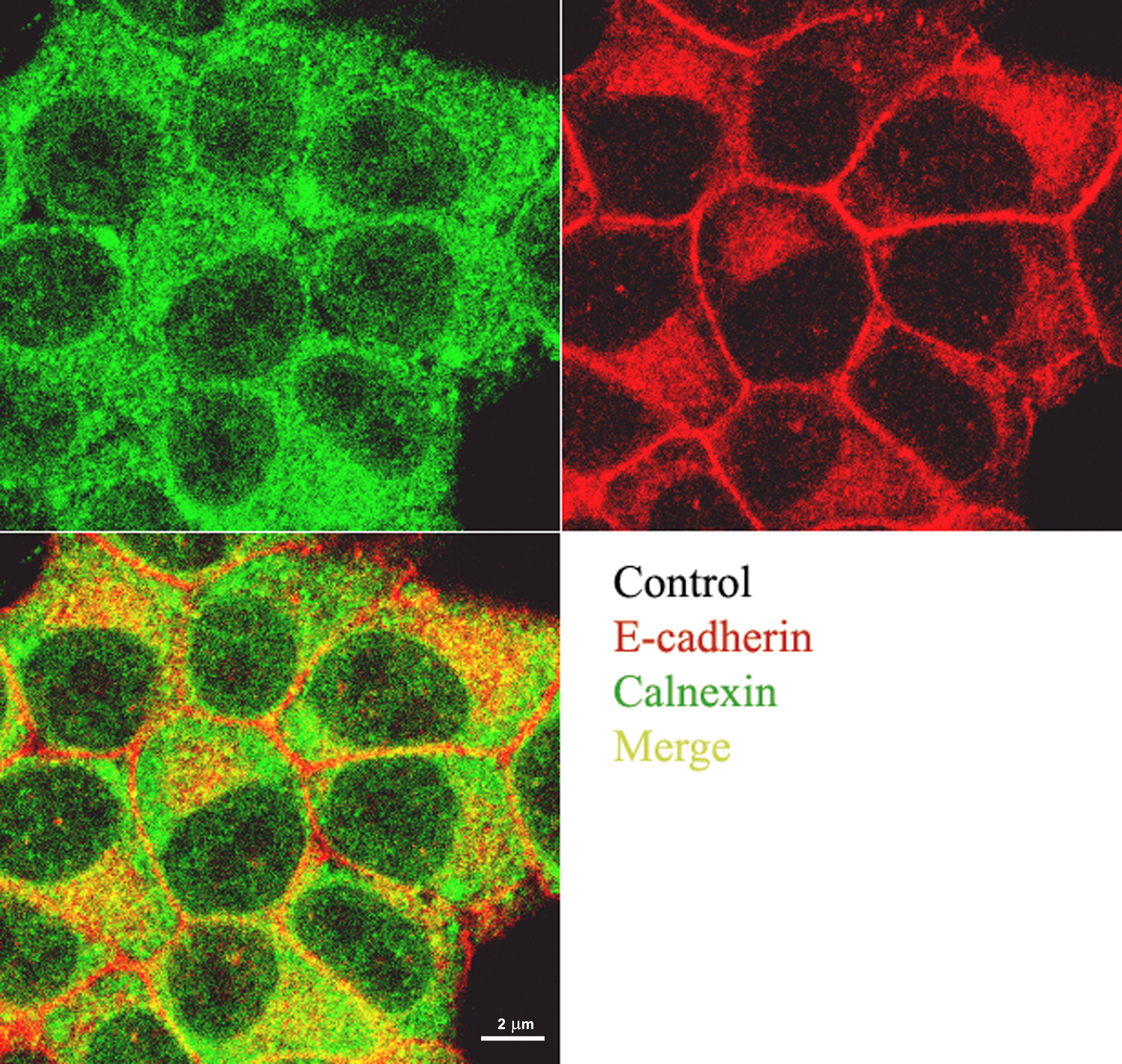

Supplement: Supplementary file 11 — Online Resource 10 [file 12020_2023_3412_MOESM11_ESM.tif]

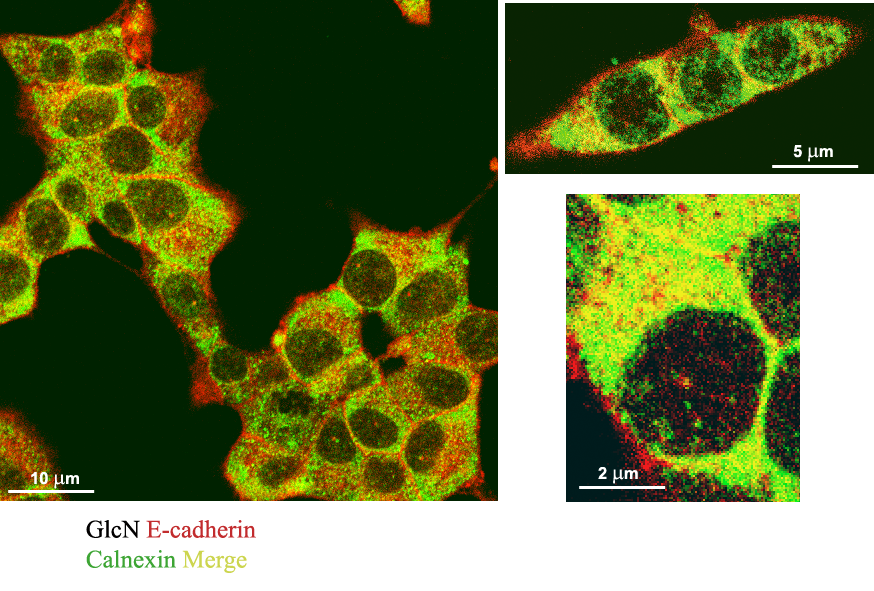

Supplement: Supplementary file 12 — Online Resource 11 [file 12020_2023_3412_MOESM12_ESM.tif]

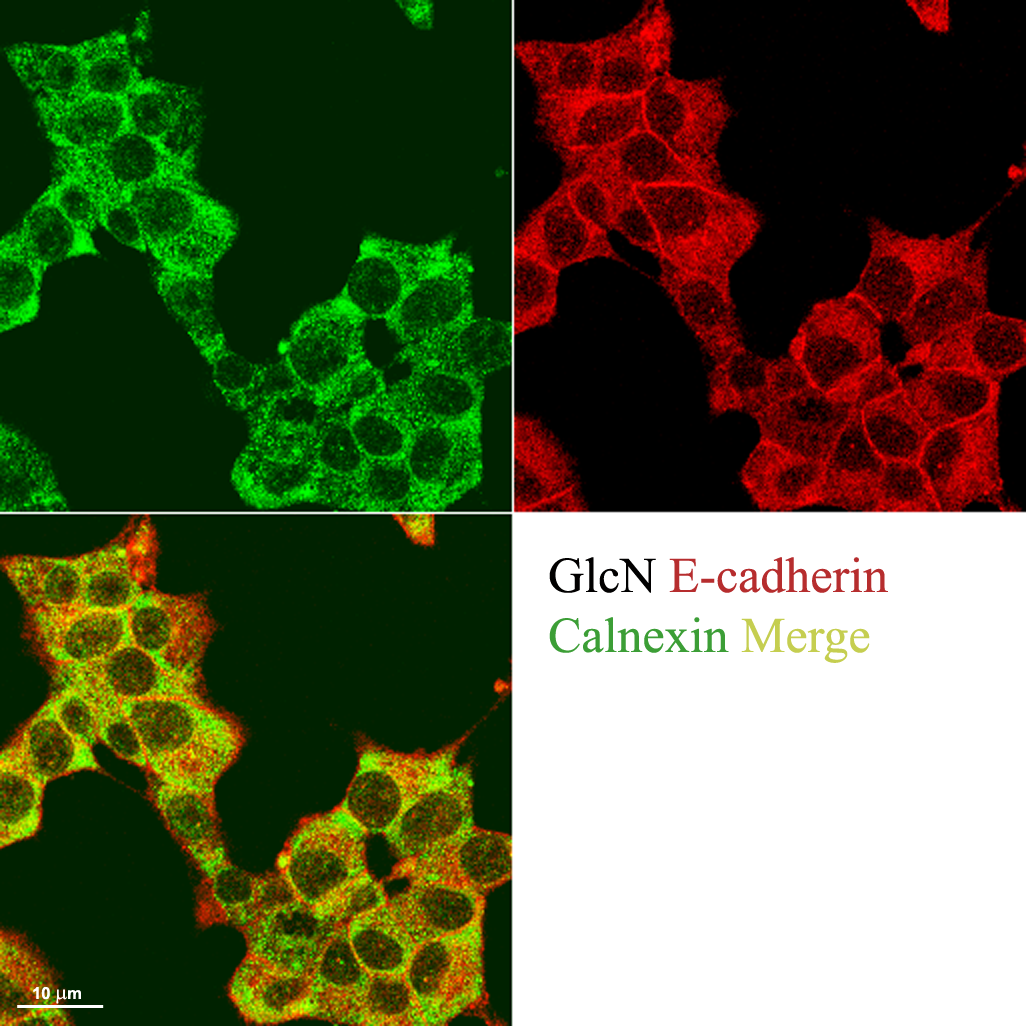

Supplement: Supplementary file 13 — Online Resource 12 [file 12020_2023_3412_MOESM13_ESM.tif]

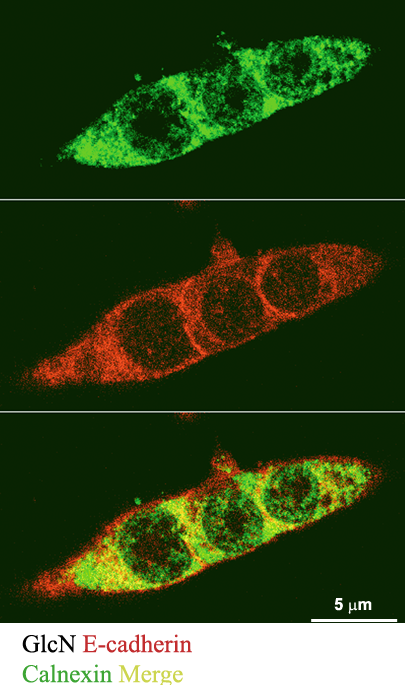

Supplement: Supplementary file 14 — Online Resource 13 [file 12020_2023_3412_MOESM14_ESM.tif]

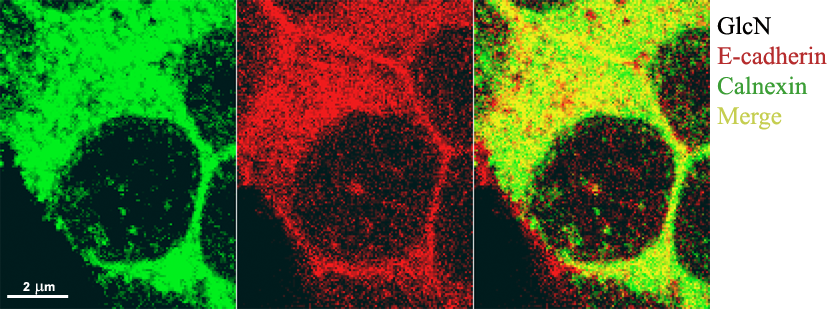

Supplement: Supplementary file 15 — Online Resource 14 [file 12020_2023_3412_MOESM15_ESM.tif]
